# Influence of Mycobiota in the Nasopharyngeal Tract of COVID-19 Patients

**DOI:** 10.3390/microorganisms12071468

**Published:** 2024-07-19

**Authors:** Veronica Folliero, Carlo Ferravante, Federica Dell’Annunziata, Rosario Nicola Brancaccio, Ylenia D’Agostino, Giorgio Giurato, Roberta Manente, Ilaria Terenzi, Rita Greco, Giovanni Boccia, Pasquale Pagliano, Alessandro Weisz, Gianluigi Franci, Francesca Rizzo

**Affiliations:** 1 Department of Medicine, Surgery and Dentistry “Scuola Medica Salernitana”, University of Salerno, 84081 Salerno, Italy; vfolliero@unisa.it (V.F.); federica.dellannunziata@unicampania.it (F.D.); manente392@gmail.com (R.M.); gboccia@unisa.it (G.B.); ppagliano@unisa.it (P.P.); 2 Laboratory of Molecular Medicine and Genomics, Department of Medicine, Surgery and Dentistry “Scuola Medica Salernitana”, University of Salerno, 84081 Salerno, Italy; cferravante@gmail.com (C.F.); rbrancaccio@unisa.it (R.N.B.); ydagostino@unisa.it (Y.D.); alessandrobianco2000@yahoo.it (G.G.); iterenzi@unisa.it (I.T.); aweisz@unisa.it (A.W.); 3 Medical Genomics Program, AOU ‘S. Giovanni di Dio e Ruggi d’Aragona’, University of Salerno, 84131 Salerno, Italy; 4 Department of Experimental Medicine, University of Campania Luigi Vanvitelli, 80138 Naples, Italy; 5 Genome Research Center for Health—CRGS, Campus of Medicine, University of Salerno, 84081 Salerno, Italy; 6 UOC Microbiologia e Virologia, AORN S. Anna e S. Sebastiano, 81100 Caserta, Italy; ritagreco67@libero.it; 7 UOC Igiene Ospedaliera ed Epidemiologia, DAI Igiene Sanitaria e Valutativa, San Giovanni di Dio e Ruggi D’Aragona, University of Salerno, 84131 Salerno, Italy; 8 Infectious Disease Unit, San Giovanni di Dio e Ruggi D’Aragona, University of Salerno, 84131 Salerno, Italy; 9 Clinical Pathology and Microbiology Unit, San Giovanni di Dio e Ruggi D’Aragona, University of Salerno, 84131 Salerno, Italy

**Keywords:** mycobiota, SARS-CoV-2, COVID-19, RNA-seq

## Abstract

The nasopharyngeal tract contains a complex microbial community essential to maintaining host homeostasis. Recent studies have shown that SARS-CoV-2 infection changes the microbial composition of the nasopharynx. Still, little is known about how it affects the fungal microbiome, which could provide valuable insights into disease pathogenesis. Nasopharyngeal swabs were collected from 55 patients, during three distinct COVID-19 waves that occurred in the Campania Region (southern Italy). An RNA-seq-based analysis was performed to evaluate changes in mycobiota diversity, showing variations depending on the disease’s severity and the sample collection wave. The phyla Basidiomycota and Ascomycota were shown to have higher abundance in patients with severe symptoms. Furthermore, the diversity of the fungal population was greater in the second wave. Conclusion: According to our research, COVID-19 induces significant dysbiosis of the fungal microbiome, which may contribute to disease pathogenesis, and understanding its underlying mechanisms could contribute to developing effective treatments.

## 1. Introduction

The microbiota plays a critical role in various aspects of host physiology, and its disruption can contribute to or directly cause diseases affecting nearby or distant organ systems [1]. While much research has focused on the bacterial component of the microbiota in both healthy and diseased states, fungal infections also represent a significant burden. However, understanding of the composition and function of the mycobiota in health and disease remains limited, largely due to its low abundance in the airways and the limited availability of detailed databases. Fungi constitute only 0.1% of the total microbiota, and as of now, only 57 complete fungal genomes have been sequenced [1,2,3]. Despite its low biomass compared with bacteria and viruses, changes in fungal composition have been observed in patients with infectious respiratory diseases. For instance, Prevel et al. identified Agaricomycetes and Ascomycota in the mycobiota of patients with ventilator-associated pneumonia [4], and Shajiei et al. demonstrated a predominance of *Candida* in the respiratory mycobiota of patients undergoing mechanical ventilation for pneumonia [5]. The coronavirus disease 2019 (COVID-19) pandemic has had profound global implications, resulting in a significant number of cases and fatalities worldwide. As reported by the World Health Organization, there have been over 670 million confirmed cases of COVID-19 and 6 million deaths across 215 countries [6]. COVID-19 patients exhibit a spectrum of clinical presentations classified as mild, moderate, or severe [7], but the factors contributing to these varying symptom severities remain poorly understood. Emerging evidence suggests that microbiome dysbiosis may play a role. Microbiome dysbiosis, characterized by imbalance in microbial communities, has been observed in patients with various viral respiratory infections, influencing the severity of clinical outcomes. Harding et al. found an enrichment of Actinomyces, Clostridiales, Lactobacillaceae, Odoribacteraceae, and S24_7 in the fecal microbiota of patients with severe/moderate respiratory syncytial virus infection [8]. Similar findings were reported by Stewart et al., who observed a correlation between streptococcal abundance and the use of positive pressure ventilation in infants hospitalized for bronchiolitis [9]. Several studies have highlighted microbiome dysbiosis in severe COVID-19 cases. Yeoh et al. reported decreased levels of gut commensals like Bifidobacteria, *Eubacterium rectale*, and *Faecalibacterium prausnitzii* in critically ill COVID-19 patients, and these are known for their potential immunomodulatory effects [10]. Additionally, Soffritti et al. identified increased levels of bacterial genera such as *Abiotrophia*, *Aggregatibacter*, *Atopobium*, *Capnocytophaga*, *Lactobacillus*, *Prevotella*, *Porphyromonas*, *Streptococcus*, and *Veillonella*, and reduced levels of others like *Rothia*, *Haemophilus*, *Parvimonas*, *Fusobacterium*, and *Gemella* spp. in the oral microbiome of severe COVID-19 patients [11]. Giugliano et al. noted alterations in the nasopharyngeal microbiome of critically ill COVID-19 patients, including reductions in Proteobacteria and increases in *Streptococcus oralis*, *Rothia mucilaginosa*, *Veillonella*, and *Prevotella* [12]. Furthermore, recent studies have expanded the scope to include viral components of the microbiome in COVID-19. Stewart et al. found associations between Human alphaherpesvirus 1 and Human gammaherpesvirus 4 with severe manifestations of COVID-19, while members of the Anelloviridae family were more prevalent in moderate cases [9]. Similarly, Ferravante et al. identified the presence of *Lymphocryptovirus* and *Simplexvirus* genera from the Herpesviridae family in critically ill COVID-19 patients [13]. SARS-CoV-2 infections lead to dysregulation of the immune response, characterized by reduced cytokine production and T-cell activity [14]. Considering the role of the mycobiota in regulating immune responses via T cells, understanding the mycobiota profile in COVID-19 patients and its variations based on infection symptoms is crucial. This knowledge could inform therapeutic strategies targeting specific secondary fungal infections and ultimately improve COVID-19 patient outcomes.

## 2. Materials and Methods

### 2.1. COVID-19 Patients

This research enrolled 55 patients from the Campania Region who were diagnosed with SARS-CoV-2 infections through molecular tests. Nasopharyngeal swabs were obtained during three distinct waves of infection: (i) March 2020 to May 2020; (ii) September 2020 to November 2020; (iii) January 2021 to February 2021. Specifically, 25 samples were attributed to the first wave, 25 to the second wave, and 5 were collected in the third wave. The samples were further subdivided according to the severity of the infection and grouped into the following categories: (i) non-serious (39 samples), if they had no symptoms and no radiographic evidence of pneumonia; (ii) moderate (6 samples), if they had pneumonia but did not require mechanical ventilation; and (iii) severe (10 samples), if they experienced respiratory failure necessitating mechanical ventilation. In total, 31% (n. 17) of recruited patients were female while their male counterparts accounted for 65% (n. 36) (Table 1). For symptomatic COVID-19 patients, nasopharyngeal swab specimens were collected immediately after symptom onset. For asymptomatic COVID-19 patients, swabs were obtained 5–7 days following close contact with an infected individual. Since samples were collected immediately after symptom onset, no patients had received antibiotics or glucocorticoids prior to sampling. Furthermore, the enrolled patients had no concomitant pathologies. Our research focused on the differences in nasopharyngeal fungemia among COVID-19 patients with varying degrees of severity (non-severe, moderate, and severe) across the three different pandemic waves. Consequently, this research did not include a healthy population for comparison. The research project was approved by the “Campania Sud” Ethics Committee (approval code: 206/2021) and was performed in accordance with the Declaration of Helsinki [15].

### 2.2. Total RNA Extraction and Quality Checks

All samples were subjected to total RNA extraction using an ELITeInGenius SP RNA cartridge (ELITechGroup, Turin, Italy) via an ELITeInGenius fully automated system (ELITechGroup). The yield of the extract was determined using the Qubit 2.0 fluorometer assay (Thermo Fisher Scientific, Waltham, MA, USA) and a 4200 TapeStation System (Agilent Technologies, Santa Clara, CA, USA). The viral presence and its load in the sample were evaluated by quantitative real-time reverse transcription PCR (qRT-PCR). A volume of 2 μL was reverse transcribed into cDNA via a SensiFAST cDNA synthesis kit (Bioline, Exalenz, UK). Thereafter, PCR amplification of a region in the SARS-CoV-2 nucleocapsid (N) gene used the following primers: forward—GGGGAACTTCTCCTGCTAGAAT and reverse—CAGACATTTTTTTTGCTCTCAAGCTG. The qRT-PCR cycle threshold values ranged from 12.18 to 34.96, which corresponded to a viral load of 1.5 × 10^7^ to 8 copies/mL, respectively.

### 2.3. Library Preparation, Sequencing, and Bioinformatic Analyses

The RNA concentration was assessed using a Qubit RNA HS Assay Kit (Thermo Fisher Scientific) and a TruSeq Stranded Total RNA Kit (Illumina, San Diego, CA, USA) for library preparation starting from 100 ng of total RNA. A Qubit dsDNA HS Assay Kit (Thermo Fisher Scientific) was used for the final library quantification, and the library size was checked with a 4200 TapeStation System. Finally, the libraries were analyzed on the NextSeq 500 (Illumina) by paired-end sequencing (2 × 75 bp). Alternatively, the NovaSeq 6000 sequencer (Illumina) was used in paired-end mode (2  ×  100 bp). In order to generate FASTQ files for downstream analysis, the de-multiplexing step was performed with Illumina bcl2fastq software v2.20. Obtained raw files were analyzed in the HOME-BIO pipeline [16]. Through the settings on the “quality control” module, reads with low quality were removed along with reads that mapped on the host reference genome (GRCh38.p13 release 37). Through querying RefSeq complete fungal genomes/proteins database obtained by the command: “kraken2-build-download-library fungi”, fungal classification was achieved. Taxonomic reports were then imported in R software (version 3.6.3) and an RPM normalization (reads per million) was finally applied. Fungal entities with fewer than three reads in half of the samples were excluded from the analysis. Subsequently, the differential distribution of phyla was calculated as the ratio of the average RPM values between the analyzed groups. Statistical significance was assessed using a *t*-test with a Bonferroni correction, considering only comparisons with a *p*-value of 0.05 or less as significant, as detailed by Harding et al. (2022) [8].

## 3. Results

### 3.1. Sequencing Quality

**Figure 1:**
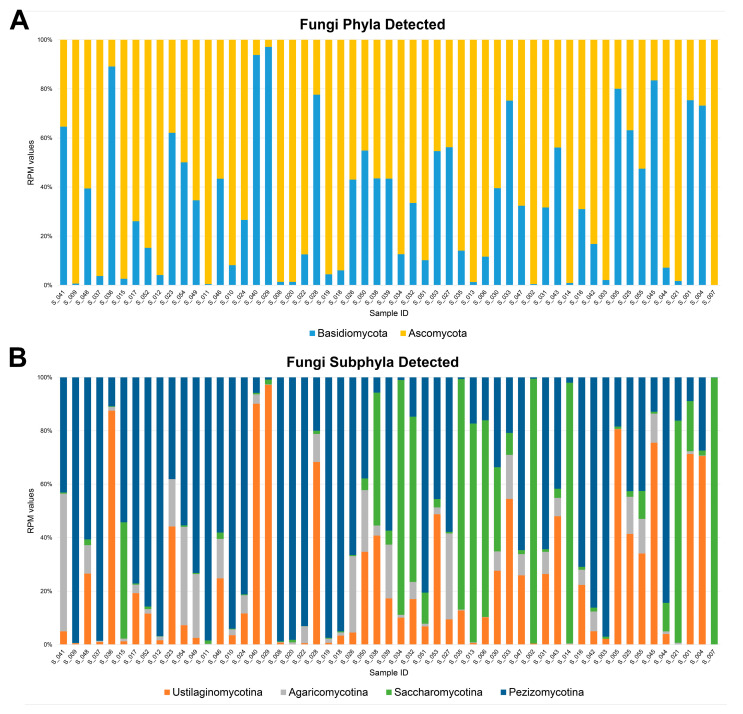
Bar plot of phyla (**A**) and subphyla (**B**) detected with a minimum of three reads in at least 50% of the 55 nasopharyngeal swab samples collected from the SARS-CoV-2-positive patients.

The NGS analysis of 55 RNA nasopharyngeal swab samples from COVID-19 patients yielded over six billion reads, with an average of 109,809,145 reads per sample. About 1.3 million reads per sample were filtered out due to low quality and the presence of adapters. In addition, 54% of the remaining reads per sample were excluded from the analysis because they mapped on the human genome, and a total of 43,184,475 reads per sample were used for downstream analysis. HOME-BIO was able to assign to fungal entities about 0.26% of all high-quality and host-filtered reads, corresponding to 3,080,769 paired fragments in the entire dataset. Totals of two and four phyla and subphyla were retained (when they were identified in at least 50% of all samples with a minimum of three reads) and considered as detected in the 55 nasopharyngeal swabs taken from the patients infected with SARS-CoV-2. Ascomycota and Basidiomycota were the identified fungal phyla, while Ustilaginomycotina, Agaricomycotina (Basidiomycota phylum), Saccharomycotina, and Pezizomycotina (Ascomycota phylum) were the observed subphyla (Figure 1A,B).

### 3.2. Fungal Diversity in the Nasal Cavity Varied with the Period and Severity of SARS-CoV-2 Infection

**Figure 2:**
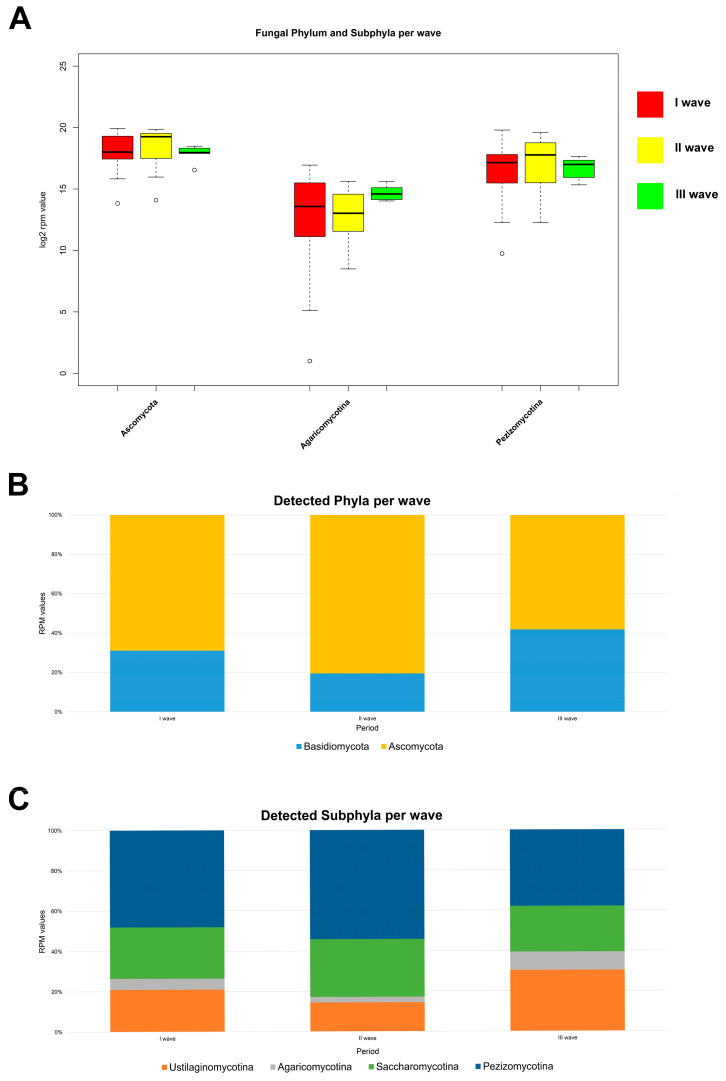
(**A**) Distribution of RPM values of fungal phyla and subphyla detected among the first (red), second (yellow), and third waves (green). Only phyla and subphyla that resulted in statistically significant association, with a *p*-value ≤ 0.05, are shown. (**B**) Relative distribution of RPM values related to detected fungal phyla and (**C**) subphyla among the three sampling periods. All fungal entities that passed the filter are shown (fungal entities were considered when they showed more than three read counts in at least 50% of samples).

**Figure 3:**
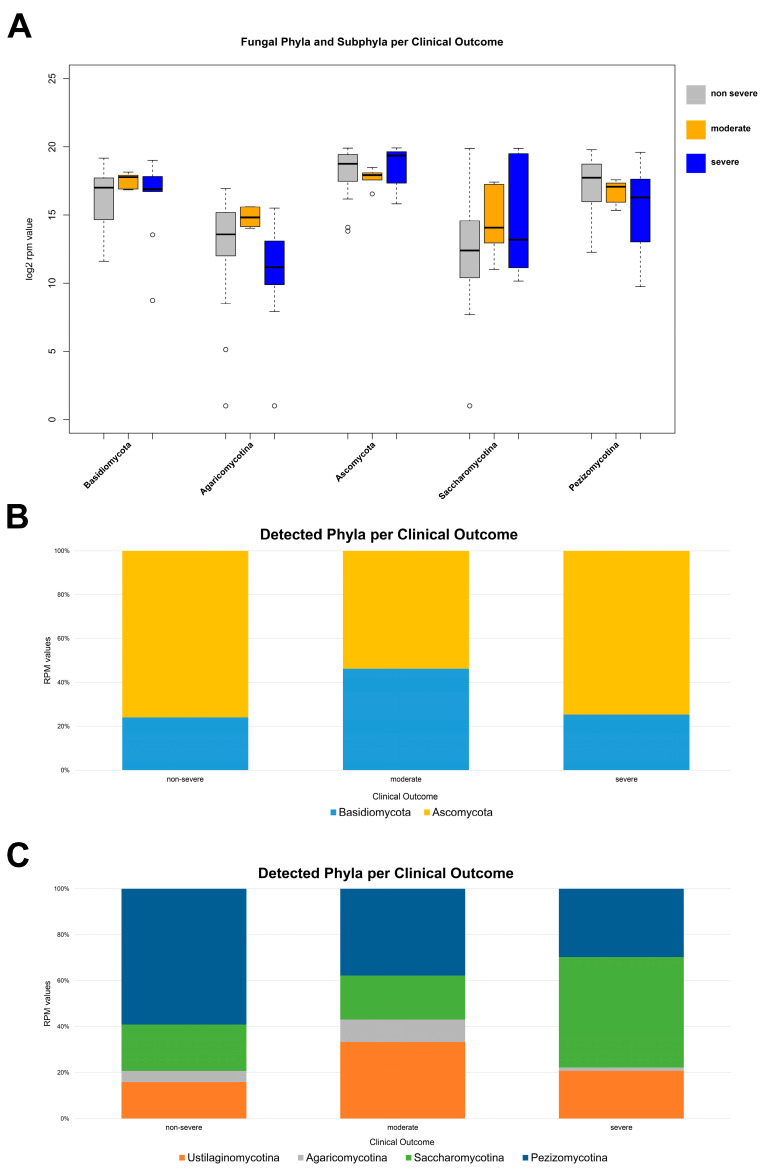
(**A**) Distribution of RPM values of fungal phyla and subphyla detected among patients with different degrees of severity: non-severe (gray), moderate (orange), and severe (blue). Only phyla and subphyla that resulted in statistically significant association, with a *p*-value ≤ 0.05, are shown. (**B**) Relative distribution of RPM values related to detected fungal phyla and (**C**) subphyla according to the severity degree. All fungal entities that passed the filter (fungal entities were reported when they show a minimum of three reads count in more than 50% of samples) are shown.

To determine whether SARS-CoV-2 infection induced a state of fungal dysbiosis in the nasopharyngeal tract, total RNA-seq technology was used to understand the mycobiota composition as a function of period and severity. With regards to the infection period, the mycobiota population of the nasopharyngeal district appeared to be more abundant in positive patients from the second wave, unlike those with infections found in the first and third periods. More specifically, the phylum Ascomycota was identified with a mean RPM value of 513,342.85 in the second wave, 398,688.90 in the first wave, and 257,430.08 in the third wave. The same trend was found for the subphylum Pezizomycotina, with mean RPM values of 279,986.65 in the second sampling wave, 230,388.31 in the first period, and 120,974.36 in the third wave of infection. A different trend was found for the subphylum Agaricomycotina, whose abundance was higher in the nasopharyngeal region of third-wave COVID-19 patients (mean RPM values of 28,965.50), followed by those positive in the first (mean RPM values of 26,140.29) and second (mean RPM values of 14,148.14) infection periods (Figure 2A,B). A relevant association between disease severity and fungal diversity was noted. The phylum Ascomycota and its subphylum Saccharomycotina were more represented in patients with prominent symptoms than in the patient group with few clinical signs, associated with mean RPM values of 541,984.91 and 310,945.34, respectively. Otherwise, the subphylum Pezizomycotina was more abundant in the patients with mild symptoms than in the group with moderate and severe clinical signs, with a mean RPM value of 274,465.86. The phylum Basidiomycota was more prevalent in patients with relevant symptoms, with mean RPM values of 205,193.72 and 184,25947 for moderate and severe COVID-19 patients, respectively; their counterparts with non-severe clinical signs were characterized by a reduced prevalence of fungi belonging to the same phylum (RPM value of 140,242.87). A reduction in the abundance of the subphylum Agaricomycotina was found in the nasopharyngeal tracts of patients with moderate and severe symptoms. Its mean RPM values were 9,300.08 for severe cases, whereas the values were 22,198.68 and 32,214.84 for mild and moderate COVID-19 (Figure 3A,B).

## 4. Discussion

COVID-19 is a highly transmissible viral infection caused by SARS-CoV-2. The disease has had a devastating global impact, resulting in over 6 million deaths worldwide. The initial cases were identified in Wuhan, Hubei Province, China, in late December 2019. The virus rapidly spread across the globe, leading the World Health Organization (WHO) to declare it a global pandemic on 11 March 2020. [6]. Despite significant advancements in clinical research that have enhanced our understanding of SARS-CoV-2, many countries continue to experience outbreaks of this viral infection. Coinfections significantly affect the clinical outcomes of COVID-19 patients. Numerous studies have extensively documented the increased risk of these infections in individuals with COVID-19, highlighting the importance of understanding the respiratory pathogens involved to improve patient management [17]. The individual effects of SARS-CoV-2 infection on host viral (viroma) and bacterial (microbiota) communities have been thoroughly investigated [18]. In contrast, limited studies have addressed how SARS-CoV-2 infection affects human mycobiota [19,20,21]. According to Zuo et al., COVID-19 patients’ faecal mycobiomes displayed changes compared with healthy controls, with an enrichment of the phylum Ascomycota [22]. Similarly, Maeda et al. demonstrated a positive correlation between SARS-CoV-2 infection and abundance of the phylum Ascomycota [23]. Furthermore, Hoque et al. reported *Saccharomyces cerevisiae* and *Aspergillus penicillioides* as the two most prevalent species in the upper respiratory tract mycobiota [24]. Based on published studies demonstrating mycobiotic alterations associated with disease presence, we hypothesized that these changes would vary based on the patient’s isolation phase and symptomatology [25]. The rapid advance of next-generation sequencing techniques, such as RNA-seq, and the implementation of fungal databases have allowed study of the global biodiversity of fungi in various regions of the body [26]. To evaluate the interaction between fungal microbiota and SARS-CoV-2 in the nasal cavities of COVID-19 patients, we analyzed RNA-seq data obtained from nasopharyngeal swabs of patients with different levels of symptom severity, collected during various pandemic waves. The nasopharyngeal tracts of positive patients belonging to the second wave of infection had a richer mycobiota population. In contrast, the reduction observed in samples from the first and third waves could be attributed to the severe restrictions imposed in the first year of the pandemic and in the post-summer periods, due to the high rates of infection. During the sample collection period, the World Health Organization reported an increase in cases from September to December, highlighting a peak of positive cases on 9 November 2021, with 242,062 cases (https://covid19.who.int/region/euro/country/it: accessed on 23 December 2021). The association between dysbiosis and the severity of SARS-CoV-2 infection is now known. Our findings indicated that COVID-19 patients with relevant symptoms had a higher representation of phylum Ascomycota, its subphylum Saccharomycotina, and phylum Basidiomycota in their nasopharyngeal tracts. Reinold et al. showed that the fungal gut microbiota in severe COVID-19 disease can be distinguished from less severe COVID-19 illness by an increase in the relative abundance of the phylum Ascomycota [27]. Hoque et al. reported a notable increase of Saccharomyces in the upper respiratory regions in COVID-19 patients compared with healthy patients [24]. Some fungal species are considered opportunistic pathogens and can cause infections when the fungal load increases. Seyedjavadi et al. showed that the main causes of co-infection were represented by species of the phylum Ascomycota, such as *Aspergillus* spp., *Candida* spp., *Pneumocystis jirovecii, Saccharomyces cerevisiae, Coccidioides* spp., *Histoplasma capsulatum,* and members of the phylum Basidiomicota, including *Cryptococcus neoformans* and *Trichosporon asahii* [28]. Among Basidiomycota, the subphylum Agaricomycotina was reduced in the nasopharyngeal tracts of patients with critical COVID-19. Agaricomycotina species are known to produce biologically active metabolites, such as polysaccharides, carbohydrate-binding proteins, peptides, and enzymes (laccase and tyrosinase), that have antiviral potential. Such bioactive compounds have been shown to inhibit viral entry and replication, as well as the expression of viral proteins [24]. Since the body’s viral load frequently directly correlates with the severity of the symptoms, its reduction leads to the alleviation of symptoms [29].

## 5. Conclusions

Overall, our results indicated a significant dysbiosis of the fungal microbiome because of SARS-CoV-2 infection, depending on restrictive practices and disease severity. Our study could be useful for improving the management of SARS-CoV-2 infection by designing mycobiota-based diagnostic practices and suitable treatment regimens, such as antifungal medications, to manage fungal co-infections in COVID-19 patients.

## Figures and Tables

**Table 1:** Features of patients enrolled in the study.

	March–May 2020	September–November 2020	January–February 2021
Age			
8–40	6	7	-
41–59	3	10	-
60–69	5	4	4
>70	8	4	1
unknown	3	-	-
Gender			
male	15	17	4
female	8	8	1
unknown	2	-	-
Disease severity			
non-severe	18	21	-
moderate	2	-	4
severe	5	4	1

## Data Availability

The original contributions presented in the study are included in the article, further inquiries can be directed to the corresponding authors.

## References

[1] Zheng D., Liwinski T., Elinav E. (2020). Interaction between Microbiota and Immunity in Health and Disease. Cell Res..

[2] Belvoncikova P., Splichalova P., Videnska P., Gardlik R. (2022). The Human Mycobiome: Colonization, Composition and the Role in Health and Disease. J. Fungi.

[3] Tang S., Jin L., Lei P., Shao C., Wu S., Yang Y., He Y., Ren R., Xu J. (2022). Whole-Genome Assembly and Analysis of a Medicinal Fungus: Inonotus Hispidus. Front. Microbiol..

[4] Prevel R., Boyer P., Beaufils F., Orieux A., Berger P., Boyer A., Delhaes L., Gruson D. (2020). The Influence of Lung and Oropharyngeal Microbiota-Mycobiota on Ventilator-Associated Pneumonia Occurrence in Critically Ill Patients: A Systematic Review. Microbiota Health Dis..

[5] Shajiei A., Liu L., Seinen J., Dieperink W., Hammerschmidt S., van Dijl J.M., Harmsen H.J.M. (2022). Specific Associations between Fungi and Bacteria in Broncho-Alveolar Aspirates from Mechanically Ventilated Intensive Care Unit Patients. Virulence.

[6] Cascella M., Rajnik M., Aleem A., Dulebohn S.C., Di Napoli R. (2023). Features, Evaluation, and Treatment of Coronavirus (COVID-19). StatPearls.

[7] Hernandez Acosta R.A., Esquer Garrigos Z., Marcelin J.R., Vijayvargiya P. (2022). COVID-19 Pathogenesis and Clinical Manifestations. Infect. Dis. Clin. N. Am..

[8] Harding J.N., Siefker D., Vu L., You D., DeVincenzo J., Pierre J.F., Cormier S.A. (2020). Altered Gut Microbiota in Infants Is Associated with Respiratory Syncytial Virus Disease Severity. BMC Microbiol..

[9] Stewart C.J., Mansbach J.M., Ajami N.J., Petrosino J.F., Zhu Z., Liang L., Camargo C.A., Hasegawa K. (2019). Serum Metabolome Is Associated With the Nasopharyngeal Microbiota and Disease Severity Among Infants With Bronchiolitis. J. Infect. Dis..

[10] Yeoh Y.K., Zuo T., Lui G.C.-Y., Zhang F., Liu Q., Li A.Y., Chung A.C., Cheung C.P., Tso E.Y., Fung K.S. (2021). Gut Microbiota Composition Reflects Disease Severity and Dysfunctional Immune Responses in Patients with COVID-19. Gut.

[11] Soffritti I., D’Accolti M., Fabbri C., Passaro A., Manfredini R., Zuliani G., Libanore M., Franchi M., Contini C., Caselli E. (2021). Oral Microbiome Dysbiosis Is Associated With Symptoms Severity and Local Immune/Inflammatory Response in COVID-19 Patients: A Cross-Sectional Study. Front. Microbiol..

[12] Giugliano R., Sellitto A., Ferravante C., Rocco T., D’Agostino Y., Alexandrova E., Lamberti J., Palumbo D., Galdiero M., Vaccaro E. (2022). NGS Analysis of Nasopharyngeal Microbiota in SARS-CoV-2 Positive Patients during the First Year of the Pandemic in the Campania Region of Italy. Microb. Pathog..

[13] Ferravante C., Sanna G., Melone V., Fromentier A., Rocco T., D’Agostino Y., Lamberti J., Alexandrova E., Pecoraro G., Pagliano P. (2022). Nasopharyngeal Virome Analysis of COVID-19 Patients during Three Different Waves in Campania Region of Italy. J. Med. Virol..

[14] Davitt E., Davitt C., Mazer M.B., Areti S.S., Hotchkiss R.S., Remy K.E. (2022). COVID-19 Disease and Immune Dysregulation. Best. Pract. Res. Clin. Haematol..

[15] Ferravante C., Arslan-Gatz B.S., Dell’Annunziata F., Palumbo D., Lamberti J., Alexandrova E., Di Rosa D., Strianese O., Giordano A., Palo L. (2022). Dynamics of Nasopharyngeal Tract Phageome and Association with Disease Severity and Age of Patients during Three Waves of COVID-19. J. Med. Virol..

[16] Ferravante C., Memoli D., Palumbo D., Ciaramella P., Di Loria A., D’Agostino Y., Nassa G., Rizzo F., Tarallo R., Weisz A. (2021). HOME-BIO (SHOtgun MEtagenomic Analysis of BIOlogical Entities): A Specific and Comprehensive Pipeline for Metagenomic Shotgun Sequencing Data Analysis. BMC Bioinform..

[17] Feldman C., Anderson R. (2021). The Role of Co-Infections and Secondary Infections in Patients with COVID-19. Pneumonia (Nathan).

[18] Candel S., Tyrkalska S.D., Álvarez-Santacruz C., Mulero V. (2023). The Nasopharyngeal Microbiome in COVID-19. Emerg. Microbes Infect..

[19] Abbasi A.F., Marinkovic A., Prakash S., Sanyaolu A., Smith S. (2022). COVID-19 and the Human Gut Microbiome: An Under-Recognized Association. Chonnam Med. J..

[20] Merenstein C., Bushman F.D., Collman R.G. (2022). Alterations in the Respiratory Tract Microbiome in COVID-19: Current Observations and Potential Significance. Microbiome.

[21] Zhang J.L., Si H.F., Shang X.F., Zhang X.K., Li B., Zhou X.Z., Zhang J.Y. (2019). New Life for an Old Drug: In Vitro and in Vivo Effects of the Anthelmintic Drug Niclosamide against Toxoplasma Gondii RH Strain. Int. J. Parasitol. Drugs Drug Resist..

[22] Zuo T., Zhan H., Zhang F., Liu Q., Tso E.Y.K., Lui G.C.Y., Chen N., Li A., Lu W., Chan F.K.L. (2020). Alterations in Fecal Fungal Microbiome of Patients with COVID-19 During Time of Hospitalization until Discharge. Gastroenterology.

[23] Maeda Y., Motooka D., Kawasaki T., Oki H., Noda Y., Adachi Y., Niitsu T., Okamoto S., Tanaka K., Fukushima K. (2022). Longitudinal Alterations of the Gut Mycobiota and Microbiota on COVID-19 Severity. BMC Infect. Dis..

[24] Hoque M.N., Rahman M.S., Sarkar M.M.H., Habib M.A., Akter S., Banu T.A., Goswami B., Jahan I., Hossain M.A., Khan M.S. (2023). Transcriptome Analysis Reveals Increased Abundance and Diversity of Opportunistic Fungal Pathogens in Nasopharyngeal Tract of COVID-19 Patients. PLoS ONE.

[25] Wu X., Xia Y., He F., Zhu C., Ren W. (2021). Intestinal Mycobiota in Health and Diseases: From a Disrupted Equilibrium to Clinical Opportunities. Microbiome.

[26] Aragona M., Haegi A., Valente M.T., Riccioni L., Orzali L., Vitale S., Luongo L., Infantino A. (2022). New-Generation Sequencing Technology in Diagnosis of Fungal Plant Pathogens: A Dream Comes True?. J. Fungi.

[27] Reinold J., Farahpour F., Schoerding A.-K., Fehring C., Dolff S., Konik M., Korth J., van Baal L., Buer J., Witzke O. (2022). The Fungal Gut Microbiome Exhibits Reduced Diversity and Increased Relative Abundance of Ascomycota in Severe COVID-19 Illness and Distinct Interconnected Communities in SARS-CoV-2 Positive Patients. Front. Cell Infect. Microbiol..

[28] Seyedjavadi S.S., Bagheri P., Nasiri M.J., Razzaghi-Abyaneh M., Goudarzi M. (2022). Fungal Infection in Co-Infected Patients With COVID-19: An Overview of Case Reports/Case Series and Systematic Review. Front. Microbiol..

[29] Choi H.M., Moon S.Y., Yang H.I., Kim K.S. (2021). Understanding Viral Infection Mechanisms and Patient Symptoms for the Development of COVID-19 Therapeutics. Int. J. Mol. Sci..

